# Opportunistic genomic screening of healthy controls in an Australian biobank

**DOI:** 10.1038/s41431-026-02104-y

**Published:** 2026-04-23

**Authors:** Lucas A. Mitchell, Mary-Anne Young, Thomas Ohnesorg, Matthew Hobbs, Joseph Copty, Jaye S. Brown, Alex W. Hewitt, Joseph E. Powell, Daniel G. Macathur, Amanda M. Willis

**Affiliations:** 1 Clinical Translation and Engagement Platform, Garvan Institute of Medical Research, Darlinghurst, NSW Australia; 2 School of Clinical Medicine, St Vincent’s Healthcare Clinical Campus, Faculty of Medicine and Health, UNSW Sydney, Sydney, NSW Australia; 3 Data Science Platform, Garvan Institute of Medical Research, Darlinghurst, NSW Australia; 4 Children’s Cancer Institute, Lowy Cancer Centre, UNSW Sydney, Kensington, NSW Australia; 5 Genomics and Inherited Disease Program, Garvan Institute of Medical Research, Darlinghurst, NSW Australia; 6 Menzies Institute for Medical Research, University of Tasmania, Hobart, TAS Australia; 7 School of Medicine, University of Tasmania, Hobart, TAS Australia; 8 Department of Ophthalmology, Royal Hobart Hospital, Hobart, TAS Australia; 9 Translational Genomics, Garvan Institute of Medical Research, Darlinghurst, NSW Australia; 10 UNSW Cellular Genomics Futures Institute, UNSW Sydney, Kensington, NSW Australia; 11 Centre for Population Genomics, Garvan Institute of Medical Research and UNSW Sydney, Sydney, NSW Australia; 12 Centre for Population Genomics, Murdoch Children’s Research Institute, Melbourne, VIC Australia

**Keywords:** Genetic counselling, Preventive medicine, Genetics research

## Abstract

Leveraging existing genomic data to opportunistically screen for secondary findings (SFs) can identify individuals at increased genetic risk who may be missed by criteria-based testing. While some guidelines support returning actionable SFs with professional support, there is a gap in consistent practice regarding the return process. This study reports the outcomes of opportunistic genomic screening in an Australian biobank. Whole genome sequencing data from 1057 healthy participants in the Tasmanian Ophthalmic Biobank (TOB), all of white European ancestry, underwent opportunistic screening for pathogenic (P) or likely pathogenic (LP) variants affecting genes in the ACMG SF v3.0 list. Variants of interest were manually curated, and only P/LP variants were returned. Actionable SFs (P/LP variants) were identified in 3.6% (38/1057) of participants. The most common genes were HFE (haemochromatosis), LDLR (Familial Hypercholesterolemia), and TP53 (Li-Fraumeni syndrome). Of the 38 participants with a variant, 27 received their result, with two-thirds being newly informed. Ten participants were referred to clinical genetics for diagnostic confirmation, while seven declined to proceed. Opportunistic screening identified a clinically significant incidence of actionable SFs in a healthy biobank cohort. There was high participant interest in receiving results, although subsequent uptake of clinical referral remains a challenge.

## Introduction

Opportunistic screening for secondary findings (SF) is increasingly a feasible way to leverage existing genomic data to identify people at genetic risk of actionable conditions and offer strategies for early detection or disease reduction. Evidence suggests opportunistic screening can identify individuals missed by traditional criteria-based testing, enabling access to proactive healthcare, such as screening, medications, or risk-reducing surgeries [1].

Previous studies have reported 1–6% of participants undergoing genomic sequencing have an actionable SF, with rates dependent on genes selected and cohort characteristics [2, 3]. Research participants report a preference to be offered SFs identified by research genomic testing [4], although there is a gap between reported preferences and actual uptake [2, 5]. Systematic reviews summarising the outcomes of receiving SFs suggest that adverse psychological outcomes are minimal [6, 7]. Some studies have demonstrated negative feelings about results and increases in distress, sometimes related to the timing and context of receiving the SF [2, 8]. However, studies also report negative feelings lessening over time and positive outcomes, including low regret, empowerment and valuing the information [2, 6, 7].

There is also support for offering actionable SFs among researchers [4, 9], and in some published guidelines and policies [10, 11]. Guidelines commonly emphasise the importance of individual choice and recommend that SFs are returned with timely support from healthcare professionals with genetic expertise to ensure SFs are appropriately managed [11]. However, there is less consistency in both guidelines and in practice regarding how and what SF to return, as well as access to genetic expertise and support [7, 11]. The penetrance of variants ascertained using a genome-first approach is also still uncertain, as much of the existing penetrance data comes from highly selected cohorts. Thus, questions remain about how to effectively and ethically return results, as well as the resources required. SF return processes must also be developed with consideration of local health systems and community attitudes.

In Australia, a national research genetic counselling platform, My Research Results (MyRR) [12], was developed to facilitate the return of results by offering guidance to researchers and providing genetic counselling directly to research participants. Here, we report on the outcomes of opportunistic screening and return of SFs performed in a partnership of MyRR with an Australian biobank of healthy controls.

## Methods

### Setting and participants

The Tasmanian Ophthalmic Biobank (TOB) is an initiative between the Royal Hobart Hospital and the University of Tasmania, set up to investigate eye disease. Participants were recruited to TOB via local newspapers, local community centres and independent living centres between January and February 2018. All TOB participants were aged 18 years and over, had no signs of ocular disease, and self-reported British, Scottish, or Irish ancestry [13, 14]. Participants provided blood samples and permission to access medical records and indicated whether they consented to being contacted with genetic results at enrolment. The TOB partnered with MyRR for opportunistic screening and return of results. Ethics approval for the study was granted by the University of Tasmania HREC, approval number H0012902.

### Whole genome sequencing and variant calling

Whole genome sequencing was performed by the Garvan Institute of Medical Research, at the time clinically accredited by the National Association of Testing Authorities, Australia (NATA) to perform whole genome sequencing analysis in accordance with the requirements of the National Pathology Accreditation Advisory Council of Australia (NPAAC) and AS ISO 15189-2013. Genomic DNA was extracted from peripheral blood lymphocytes using standard protocols, and libraries were prepared using the KAPA Hyper PCR-free Library Preparation Kit (Roche). The libraries were sequenced on an Illumina NovaSeq 6000 sequencer to produce paired-end 150 bp sequences to a mean genome coverage of at least 30X. Sequences were aligned to the GRCh38 reference genome with bwa mem v0.7.15 [15], and variants called with GATK v3.7 following GATK best practices [16].

### Variant annotation and filtering

A bioinformatics pipeline was developed to annotate and filter variants and identify candidate variants for manual interpretation. Variants in VCF format were annotated with the following tools: i) Ensembl VEP v103 [17] for variant effect predictions; ii) slivar v0.2.3 [18] for “popmax” allele frequencies in GnomAD v3 genomes [19]; vcfanno v 0.3.2 [20] for various fields derived from a Clinvar VCF file (versioned 2021-08-28) retrieved from the NCBI’s ftp site; bcftools v1.11 [21] for scores from v1.3 of REVEL [22].

Candidate variants were filtered by bcftools and included if they were in genes listed in the American College of Medical Genetics and Genomics (ACMG) SF list of minimally reportable genes (version 3.0) [23] and they satisfied any of the following criteria: i) Clinvar classification of pathogenic (P) or likely pathogenic (LP); ii) regarded as “impactful” (loss-of-function or variants with a REVEL score of ≥0.7 or spliceAI score of ≥0.2) and with gnomAD v3 grpmax FAF ≤ 0.02; iii) HFE C282Y. Variants previously observed and classified as not reportable were disregarded. Candidate variants identified in participants who had consented to genetic results proceeded to interpretation.

### Variant interpretation and reporting

Variants were curated according to ClinGen Variant Curation Expert Panel or CanVIG UK guidelines if available, or the ACMG and the Association for Molecular Pathology (AMP) variant classification guidelines by variant curator TO [24]. P/LP and suspicious variants of uncertain significance (VUS) were reviewed by a multidisciplinary committee, which included clinical geneticists, a genetic pathologist, genetic counsellors and variant curators. Variant interpretation was primarily based on genomic data only. Clinical information was not always available, given the secondary nature of the analysis. Only P/LP variants were approved for return to participants.

### Return of results

The TOB team re-identified participants where P/LP variants were identified and reviewed medical records to check the vital status of participants. Participants were notified of available research results by letter from the TOB team and directed to contact a TOB representative for more information. Participants who did not respond to the letter were followed up by telephone.

Participants who responded to the letter or phone call had the option to discuss their results with a MyRR genetic counsellor or TOB clinician. Participants who were successfully contacted by phone could choose whether to receive their results and results were disclosed according to participant preference. Participants who received their results were advised that results should be confirmed on an independent sample in a diagnostic laboratory and were supported to obtain a referral to their local clinical genetics service for diagnostic testing and clinical assessment. MyRR and TOB case files were reviewed, and data were extracted for a descriptive analysis of the outcomes of this process.

## Results

A total of 1057 participant samples underwent opportunistic screening. The average age of participants was 64 (range 19–97), the majority were over the age of 60 (73%), and the majority were female (58%).

### Variants identified

Eighty-six participants (8%) had a potential variant of interest. After preliminary file review, seven were deceased and five had not consented to receive SFs, so these variants did not proceed to interpretation. Seventy-three variants were curated and discussed by a multidisciplinary committee, with 38 classified as P (*n* = 17) or LP (*n* = 21) (Table 1). The incidence of reportable SFs from opportunistic screening in this cohort was 3.6% (38/1057). The average age of participants with a P/LP variant was 64 (range 24–87), and 20 of the 38 (53%) were female. The most common genes reported were *HFE* - Hereditary haemochromatosis (*n* = 9), *LDLR* – Familial Hypercholesterolemia (*n* = 4) and *TP53* – Li Fraumeni syndrome (*n* = 4). See Table 1 for the full list of genes reported.

**Table 1:** Number of participants with pathogenic/likely pathogenic variants identified, notified results were available and received results by gene, with reason participants were not notified of or did not receive results included.

Phenotype *gene*	P/LP variants identified (*n*)	Notified results available (*n*)	Received results (*n*)	Reason not reported/returned (*n*)
Cancer				
*TP53*	4	2	2	Deceased (2)
*PMS2*	3	3	3	N/A
*BRCA2*	1	1	1	N/A
*MSH6*	2	1	1	Deceased (1)
Cardiovascular				
*LDLR*	4	4	3	Could not contact (1)
*TTN*	3	3	3	N/A
*KCNQ1*	2	2	2	N/A
*FBN1*	2	1	1	Medical record (1)
*MYH7*	2	1	1	Medical record (1)
*FLNC*	1	1	1	N/A
*DSG2*	1	1	1	N/A
*TGFBR1*	1	1	1	N/A
Metabolic				
*HFE*	9	5	5	Deceased (2); Medical record (2)
*RYR1*	2	1	1	Deceased (1)
*OTC*	1	1	1	N/A
Total	38	28	27	

### Return of results

**Fig. 1:**
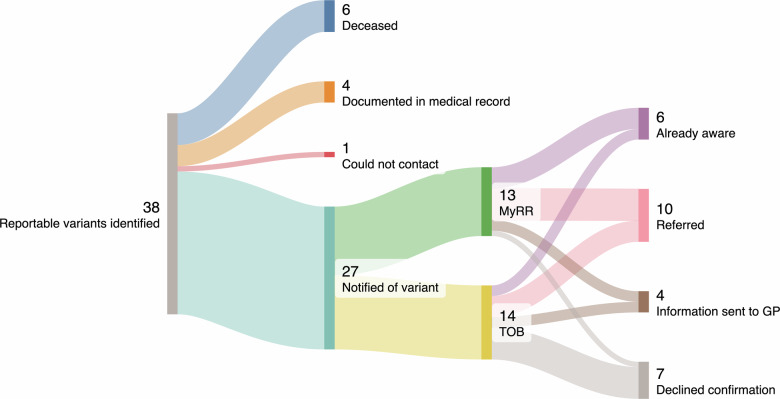
Outcomes for the 38 reportable variants identified by opportunistic screening.

The clinical outcomes for the 38 reportable variants are represented in Fig. 1. At re-identification, an additional six participants were deceased and four had the genetic result recorded in their hospital electronic medical record and were not notified of the research result. Notification letters were sent to the remaining 28 TOB participants. One participant with an *LDLR* variant could not be contacted after multiple attempts. Twenty-seven participants were successfully contacted and chose to receive their research results, 14 from the TOB clinician and 13 from a MyRR genetic counsellor.

Of the participants who received their results, six participants with results for Familial Hypercholesterolemia (*LDLR*, *n* = 2) or Hemochromatosis (*HFE*, *n* = 4) already had a genetic diagnosis and associated clinical diagnosis. Ten of the participants notified of results were referred to clinical genetics for diagnostic confirmation of the result, four opted to have the information sent to their primary care practitioner for discussion, and seven declined to proceed with any clinical confirmation (see Table 2 for outcomes with gene information).

**Table 2:** Outcomes of reportable variants with gene information.

Outcome	Gene	Total
Not notified		
Deceased	*TP53* (2)*, MSH6, HFE* (2)*, RYR1*	6
Documented in medical record	*FBN1, MYH7, HFE* (2)	4
Notified		
Could not contact	*LDLR*	1
Already aware of variant	*LDLR* (2)*, HFE* (4)	6
Information sent to PCP	*FLNC, KCNQ1, TP53, MYH7*	4
Declined clinical confirmation	*DSG2, TTN, KCNQ1, HFE, PMS2, RYR1, OTC*	7
Referred to genetics	*BRCA2, FBN1, LDLR, MSH6, PMS2* (2)*, TGFBR1, TP53, TTN* (2)	10
Total		38

*PCP* Primary care practitioner.

## Discussion

Here, we report the outcomes of opportunistic genomic screening and return of SFs to Australian biobank participants. Actionable SFs were identified in 3.6% of participants, which is consistent with SF incidence reported elsewhere [2, 3]. Almost three quarters of variants had not previously been identified, demonstrating that opportunistic screening can identify potentially high-risk variants undetected by existing models of genetic testing.

Interest in receiving SFs among participants in this biobank was high. Only five out of the 86 (6%) participants with potential variants of interest had declined to receive results at enrolment to the biobank. This is comparable to active SF decline rates in other settings, although other studies have indicated higher non-response rates [1, 25, 26]. Reasons for declining SFs were not collected in this study. Other studies report reasons for decline including not wanting to know, concerns that the information may be upsetting, perceiving the information as not applicable, insurance concerns and privacy concerns [25, 26]. Further exploration of reasons for decline in the context of local health care systems may help identify barriers to uptake.

While interest was high, only half of the eligible participants were referred, or intended to be referred, for confirmatory testing as recommended before using the result to guide clinical care. We observed higher uptake of referral among participants who received results from a genetic counsellor, although this observation is limited by the small numbers, which has been reported elsewhere [6]. Genetic counsellors play a pivotal role in healthcare, helping people understand genomics and the accompanying medical, familial, psychological and financial implications [27, 28]. Genetic counsellors also play a role in upskilling and supporting non-genetic health professionals in varied settings [27, 28]. We suggest that genetic counsellors’ core skills can be leveraged to enable more scalable, accessible and supportive provision of SFs, especially as the volume of genetic testing and SFs increases.

Overall, two thirds of TOB participants who received a new result were either referred to clinical genetic services, or had the information sent to their primary care practitioner. However, we were unable to ascertain whether participants attended a genetics appointment, the outcomes of information provision to primary care, or reasons for declining diagnostic testing. Other studies have reported low uptake of genetic visits and underutilisation of health resources after the return of research results [2, 5]. Further research into reasons for non-uptake of health services after receipt of SFs could identify barriers and aid in developing strategies to facilitate appropriate care. Longer-term follow-up is also required to capture the impact of SF return fully.

Challenges experienced through this process echo those reported in other studies. The translational nature of returning SFs requires managing clinically actionable information in a research setting, with research timelines. Extended periods may have passed since participants enroled in research, and participants may not recall consenting, have changed contact details, or had relevant diagnoses in the intervening period. Being unable to return SFs because participants are deceased, can’t be contacted, or did not consent, can cause ethical dilemmas for researchers and clinicians involved. Returning SFs to a nominated next of kin when participants are deceased is possible, but presents its own challenges, and there is limited data on this practice for SFs [9]. Deciding how far to go to contact people can also be difficult in the context of low rates of active decline and data suggesting non-response is not necessarily refusal [3, 25]. Consent models that incorporate dynamic consent and ongoing participant engagement may help to mitigate some of these logistical challenges.

While provision of SFs leverages existing genomic data, it is still resource intensive, requiring careful consideration of which SFs to offer, considered consent processes, variant curation, genetic counselling and support [9, 29]. Limited or no access to phenotype and family history information (e.g. as phenotype specificity or co-segregation evidence) [24], as was the case here, can create challenges when curating variants and interpreting results. There is also some uncertainty regarding the penetrance of variants identified by this genome-first approach [9]. This led us to take a conservative approach when determining which results to return, to minimise the potential for unnecessary worry and use of healthcare resources. However, the identification of variants using this genome-first approach can also provide opportunities to better understand the penetrance and variability of genetic disease.

All of these factors currently impact feasibility in the research setting, where funding is often scarce. Return of SFs also requires support from health systems for the long-term management of people identified at potentially high-risk, which may not have the infrastructure or resources to take on this additional workload. Despite this, researchers, clinicians and participants/patients still value the return of SFs and consider it a worthwhile endeavour [4, 9]. Identifying ways to improve the scalability of SF provision, through novel use of technology and innovative return pathways, is recommended.

### Limitations

While the outcomes from return of SFs to TOB participants are similar to those reported elsewhere, participants all had white European ancestry and were from a single region of Australia, which may limit the generalisability of the findings. Data regarding longer-term outcomes, including confirmation of research SFs and other health actions taken, were not available due to resource constraints. However, such data are important for understanding the broader impact of returning SFs and the penetrance of variants identified by this genome-first approach.

## Conclusion

This study demonstrates the feasibility of opportunistic genomic screening in a large biobank of healthy Australian controls. A significant majority of SFs were new to the participants, demonstrating that this “genome-first” approach can identify individuals missed by current symptom or family-history-based testing criteria. High participant interest in receiving these SFs was observed. However, challenges persist, including uncertainties regarding penetrance, managing the logistics of recontacting participants and the uptake of appropriate health actions. Future efforts must focus on improving the scalability of SF provision through innovative pathways, capture of longer-term data to inform penetrance and research to identify and address barriers to participants accessing recommended risk management strategies.

## Data Availability

Study data sets have not been deposited in a public repository because of consent restrictions and the sensitive nature of the data. Requests to access deidentified data that support the findings of this study can be sent to the corresponding author.
